# Comparative Population Genetics of the Immunity Gene, *Relish*: Is Adaptive Evolution Idiosyncratic?

**DOI:** 10.1371/journal.pone.0000442

**Published:** 2007-05-16

**Authors:** Mia T. Levine, David J. Begun

**Affiliations:** Center for Population Biology, University of California at Davis, Davis, California, United States of America; Indiana University, United States of America

## Abstract

The frequency of adaptive evolution acting on common loci in distant lineages remains an outstanding question in evolutionary biology. We asked whether the immunity factor, *Relish*, a gene with a history of directional selection in *Drosophila simulans*, shows evidence of a similar selective history in other *Drosophila* species. We found only weak evidence of recurrent adaptive protein evolution at the *Relish* locus in three sister species pairs, suggesting that this key component of the insect immune system has an idiosyncratic evolutionary history in *Drosophila*.

## Introduction

Convergent phenotypic evolution, which results from similar selection pressures in independent lineages, is a common, undisputed property of animal and plant evolution. The frequency of convergent adaptive *molecular* evolution, however, remains an open question. Convergent adaptive molecular variants may include both amino acid polymorphism [1]–[6] and amino acid divergence [7]–[9]. The relatively few examples of such convergent changes are based largely on molecular and functional analysis of proteins with well-defined structures and functions; consequently, the inference of convergent adaptive polymorphism or divergence allows plausible arguments to be made regarding the phenotypic adaptive effects of particular mutations in different lineages. Most gene products are not understood sufficiently well to use such an approach.

An alternative, statistical approach is to use molecular population genetic data to ask whether directional selection is repeatable over evolutionary time. For example, the McDonald-Kreitman test [10], which uses contrasts of polymorphic and fixed variants to test the neutral model of molecular evolution, requires no knowledge of protein structure or specific functions of residues or domains. This allows one to ask the general question of whether a gene with a history of recurrent adaptive protein evolution in one species is likely to have a similar selective history in other species; that is, is directional selection idiosyncratic or predictable? For example, the Drosophila seminal fluid protein gene *Acp26Aa* was first inferred to have a history of recurrent adaptive protein evolution in the *melanogaster* subgroup [11]. *Acp26Aa* was later shown to be under such selection in the *obscura* group of *Drosophila* [12], which diverged from the *melanogaster* subgroup tens of millions of years ago.

The *Drosophila* innate immune system transcription factor, *Relish*, is a potentially interesting gene for addressing the question of predictable versus idiosyncratic directional selection. Previous studies demonstrate that the innate immune system, a highly conserved pathway from insects to humans, is vulnerable to signaling disruption by both bacterial and viral pathogens. Moreover, Relish activation and/or signaling repeatedly emerges as a pathogen target. In a vertebrate system, Neish et al. [13] demonstrate that *Yersina* bacteria disrupts phosphorylation of the human Relish homolog, NF-κB. In an insect system (*Drosophila melanogaster*), Lindmark et al. [14] and Thoetkiattikul et al. [15] demonstrate Relish signaling disruption by various bacteria and a polydnavirus, respectively.

Compromised immune response in the presence of these pathogens, combined with documented Relish-pathogen interactions, makes this locus a likely target for repeated host-pathogen evolutionary interactions in distantly related taxa. Nevertheless, population genetic data for the Relish locus provided strong evidence of adaptive divergence in *D. simulans*, but no evidence of adaptive divergence in *D. melanogaster* [16]. Similarly, the termite *Relish* locus appears to be rapidly evolving in a subset of lineages [17]. Although *Relish* likely contributes to immune function in all species examined, the evolutionary dynamics associated with this locus are dramatically different across lineages. To further investigate the repeatability of directional selection at this locus in *Drosophila*, we characterized the evolutionary forces acting on *Relish* across three highly diverged sister species-pairs, *D. mojavensis/D. arizonae, D. yakuba/D. teissieri*, and *D. pseudoobscura/D. miranda*.

## Results and Discussion

Levels of synonymous and nonsynonymous polymorphism at *Relish* (Table 1) were consistent with previous descriptions *Drosophila mojavensis*/*D. arizonae* [18], whereas lower than expected levels of variation were estimated for *D. yakuba* [19] and *D. pseudoobscura* [20]. Levels of *Relish* synonymous divergence in these species pairs were typical of those estimated at other genes. Levels of non-synonymous divergence (scaled to synonymous divergence), however, were highly heterogeneous across species, suggesting the protein evolutionary rates vary due to heterogeneous selection regimes (Table 1).

**Table 1:** Polymorphism and divergence for all species examined as well as previously published estimates for *D. melanogaster* and *D. simulans*.

**Polymorphism**				
Species	# lines	#sites	π (syn.)	π (nonsyn.)
*D. melanogaster*	6	2801	0.036	0.022
*D. simulans*	7	2801	0.062	0.029
*D. yakuba*	8	2303	0.0034	0.0
*D. tessieri*	5	2303	0.042	0.0019
*D. mojavensis*	6	2792	0.018	0.0012
*D. arizonae*	6	2792	0.015	0.0012
*D. pseudoobscura*	6	2191	0.0062	0.00065
*D. miranda*	1	2191	n/a	n/a
**Divergence**				
Species Pair	*Ks* *	*Ka* *	*Ka*/*Ks*	
*D. mel/D. sim*	0.099	0.052	0.53	
*D. yak/D. teiss*	0.088	0.0044	0.05	
*D. moj/D. ariz*	0.062	0.0064	0.10	
*D. pseudo/D. mir*	0.057	0.017	0.30	

* Ka and Ks refer to the nonsynonymous and synonymous substitution rates, respectively.

We used the McDonald-Kreitman test to determine whether synonymous and non-synonymous variation at *Relish* supports the hypothesis of adaptive protein evolution. All three species pairs failed to reject the null hypothesis of neutral evolution (Table 2). The *D. simulans/D. melanogaster* species pair is the only one associated with evidence of adaptive protein evolution at *Relish* [16].

**Table 2:** McDonald-Kreitman tests of *Relish* variation for four species pairs.

	Synonymous		Nonsynonymous		
Species	Fixed	Polymorphic	Fixed	Polymorphic	G (p-value)
*D. yak/D. teiss*	28	50	5	7	0.15 (0.70)
*D. pse/D. mir*	24	7	24	3	1.37 (0.24)
*D. moj/D. ariz*	14	44	7	13	0.86 (0.35)
a *D. mel/D. sim*	40	41	89	10	37.5 (<10^−4^)

a [16]

Low levels of polymorphism at *Relish* in *D. yakuba* and *D. pseudoobscura* could be due to recent, strong directional selection at *Relish* or at linked sites. We used the HKA test [21] to determine whether the polymorphism-to-divergence ratios at *Relish* were unusual compared to those from the putatively neutral loci *Xdh* in *D. yakuba/D. teissieri*, (J. Comeron pers. comm.) and *Adh* in *D. pseudoobscura/D. miranda* [22]. Only the *D. yakuba/D. teissieri* data rejected the null (*χ* ^2^ = 6.39, *p* = 0.01), which is consistent with linked selection in this region of the *D. yakuba* genome. The *Relish* gene is near the middle of chromosome arm 3R in *D. yakuba* (*D. yakuba* genome assembly, v2), which suggests that this result is not due to sampling a large region of reduced polymorphism near centromeres and telomeres [23]. Further analysis of the regions flanking *Relish* is necessary to determine the extent of reduced polymorphism in this genomic region.

The Relish population genetic data from three, distantly related, *Drosophila* species pairs generally supports the idea that Relish evolution in the *D. melanogaster/D. simulans* pair is highly unusual. Previous analyses of D. *melanogaster/D. simulans* suggest that evidence of strong directional selection at *Relish* is most likely a *D. simulans*-lineage phenomenon [16]. This finding raises the interesting question of what *D. simulans*-specific biological or historical attributes caused the highly unusual history of a key component of the insect immune system.

## Methods

Population samples of *Relish* were sequenced from inbred lines of *D. yakuba* (P. Andolfatto), *D. tessieri* (M. Long), *D. mojavensis* (W. Etges and Tucson Stock Center), *D. arizonae* (W. Etges), *D. pseudobscura* (M. Noor), *D. miranda* (Tucson Stock Center). Most data were obtained by direct sequencing. For the few lines with residual heterozygosity, PCR products were cloned in PCR-4 vector (Topo TA cloning kit, Invitrogen) and individual colonies were sequenced. Population genetic estimators and tests statistics were calculated in DnaSP v.4.0 (Rozas et al. 2003). Sequence data for this paper have been submitted to Genbank under accession numbers EF494515-EF494539.
